# Brain tumor detection from images and comparison with transfer learning methods and 3-layer CNN

**DOI:** 10.1038/s41598-024-52823-9

**Published:** 2024-02-01

**Authors:** Mohammad Zafer Khaliki, Muhammet Sinan Başarslan

**Affiliations:** 1 Ataşehir Bil Anatolian High School, 34720 Istanbul, Turkey; 2 Department of Computer Engineering, Faculty of Engineering and Natural Sciences, Istanbul Medeniyet University, 34885 Istanbul, Turkey

**Keywords:** Biomedical engineering, Cancer

## Abstract

Health is very important for human life. In particular, the health of the brain, which is the executive of the vital resource, is very important. Diagnosis for human health is provided by magnetic resonance imaging (MRI) devices, which help health decision makers in critical organs such as brain health. Images from these devices are a source of big data for artificial intelligence. This big data enables high performance in image processing classification problems, which is a subfield of artificial intelligence. In this study, we aim to classify brain tumors such as glioma, meningioma, and pituitary tumor from brain MR images. Convolutional Neural Network (CNN) and CNN-based inception-V3, EfficientNetB4, VGG19, transfer learning methods were used for classification. F-score, recall, imprinting and accuracy were used to evaluate these models. The best accuracy result was obtained with VGG16 with 98%, while the F-score value of the same transfer learning model was 97%, the Area Under the Curve (AUC) value was 99%, the recall value was 98%, and the precision value was 98%. CNN architecture and CNN-based transfer learning models are very important for human health in early diagnosis and rapid treatment of such diseases.

## Introduction

The healthcare industry has been rapidly transformed by technological advances in recent years, and an important component of this transformation is artificial intelligence (AI) technology. AI is a computer system that simulates human-like intelligence and has many applications in medicine. One such area is the fight against brain tumors. Brain tumors are a major public health problem in the healthcare sector, and accurate diagnosis, treatment, and follow-up processes are critical. AI has become an important tool for improving these processes and has great potential for early diagnosis and treatment of brain tumors.

Brain tumors affect human health due to their location^1^. AI is designed to help diagnose and treat complex diseases such as brain tumors by combining technologies such as big data analytics, machine learning, and deep learning. AI has the ability to detect and classify tumors by analyzing brain imaging techniques, such as Magnetic Resonance Imaging (MRI). AI algorithms can help determine the size, location, class, and aggressiveness of tumors. This helps physicians make a more accurate diagnosis and treatment plan, and helps patients better understand their health.

AI can also be used to track a patient's progress through treatment. AI-based analytics can be used to assess treatment response and predict potential tumor recurrence. In this way, patients' treatment plans can be more effectively organized and individualized treatment approaches can be developed.

In this study, difference detection was performed on brain images. Classification was performed with multilayer CNN and CNN-based transfer learning methods on 4 classes labeled by physicians.

The contribution of the study is as follows.

- We investigate the transfer learning method with the highest performance in the classification process of transfer learning methods on brain images.
- We investigate the performance of CNN and transfer learning on brain images using CNN as a multi-layer without using transfer learning.
- We investigate whether it is possible to achieve good results with a skewed and poor quality dataset.

**Figure 1:**
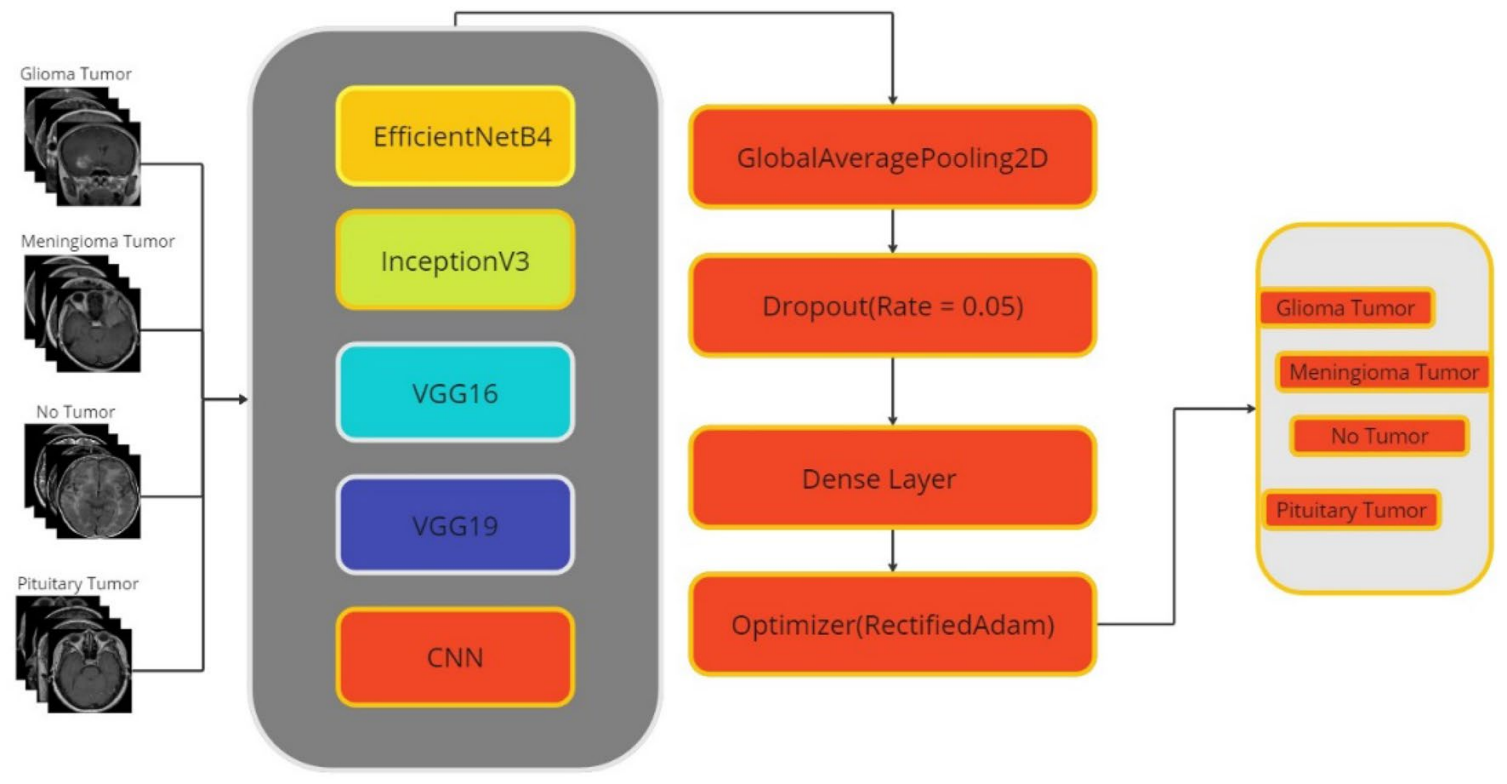
Flow diagram of the work.

The flow diagram of the study is shown in Fig. 1.

Studies on brain tumors in the last 5 years will be scanned from indexes such as WOS and IEEE and the details of the related studies will be explained in this section.

In a study of 3064 MRI images from 233 patients belonging to 4 different tumor classes (meningioma, glioma, pituitary, tumor) with support vector machine (SVM) on datasets generated after various preprocessing, an accuracy value between 94% was obtained^1^. Santhosh and colleagues presented a classification model aimed at distinguishing between normal and abnormal brain tissue. This system relied on a combination of thresholding and watershed segmentation techniques. Using SVM, the classification accuracy reached an impressive 85.32% across all categories^2^. Rafael et al. achieved an accuracy rate of 89.6% using SVM for brain tumor classification^3^. Similarly, Gupta and Sasidhar achieved 87% accuracy using SVM^4^. Gumaei et al. proposed a classification framework that harnesses the power of regularized extreme learning machine (RELM) for the purpose of distinguishing between benign and malignant brain tumors. Their study involved the collection and preprocessing of MRI data related to meningioma, glioma, and pituitary tumors. The feature selection process was performed using GIST, Normalized GIST (NGIST), and PCA-NGIST methods. Using a meticulous fivefold cross-validation procedure, the RELM technique yielded an impressive overall accuracy of 92.61%^5^. 92% accuracy was achieved using SVM machine learning on a dataset of 90 normal and 154 tumor brain images^6^. 3264 brain tumor images from Kaggle were classified using CNN, LSTM and CNN-LSTM hybrid. The results obtained; CNN 89%, LSTM 90.02%, CNN-LSTM 92% accuracy^7^. Srinivas and co-authors conducted a comprehensive study involving a comparative performance analysis of transfer learning based CNN models pre-trained with VGG16, ResNet-50 and InceptionV3 architectures for brain tumor cell prediction. In particular, InceptionV3 had an accuracy of 78%, VGG16 had a high accuracy of 96%, and ResNet-50 had an accuracy of 95%^8^. In a CNN-based study of brain tumor images, Choudhury, Mahanty, Kumar, and Mishra achieved an accuracy of 96.08%^9^, while Martini and Oermann's CNN-based study achieved an accuracy of 93.9%^10^. Between 2005 and 2010, a study was conducted to predict the classes of meningioma, glioma, and pituitary gland from brain images of 233 patients in China. In this study, 4-layer CNN was used. The accuracy was 91.3%^11^. In a study of 200 brain tumor images, the accuracy of image segmentation was 92.14%^12^. Classification studies on 102 brain tumor patients using SVM and KNN machine learning methods achieved 85% and 88% accuracy, respectively^13^. In a classification study of 233 brain tumor patients, SVM and KNN were used. In this study, the accuracy result was 91.28%^14^. In a classification study using CNN on 233 patient images with meningioma, glioma or pituitary tumor, the accuracy was 91.43% with fivefold cross-validation^15^. The author introduced a novel approach known as a Capsule Network (CapsNet), which effectively integrates brain MRI images and approximate tumor boundaries for the purpose of brain tumor classification. This study achieved an impressive accuracy of 90.89% in accurately classifying brain tumors^16^. In this study, as seen in the literature, CNN and CNN-based transfer learning methods will be used for brain tumor detection.

## Material method

This section describes the dataset, the classification algorithm (CNN) used in the study, and the transfer learning architectures VGG19, VGG16, InceptionV3, EfficientNetB4 developed based on this algorithm.

### Data source

The dataset consists of a total of 2870 human brain MRI images systematically classified into four different categories: glioma, meningioma, no tumor and pituitary. The distribution of labeled images into these four classes is shown in Table 1 for reference^17^.

**Table 1:** Distribution of the preprocessed brain tumor dataset.

Data	Glioma	Meningioma	No tumor	Pituitary	Total
Training data	696	704	316	676	2452
Testing data	92	93	49	90	324
Validation data	138	140	75	135	488

Glioma is the most common type of malignant brain tumor and typically occurs in glial cells in the brain and spinal cord. Meningioma is a benign type of brain tumor, but can become malignant without appropriate intervention. These classes are labeled by physicians. The size of the input images is 64 × 64. Table 1 shows the training, test and validation set discriminations by class.

### Deep learning

Deep learning is a subset of machine learning that focuses on training artificial neural networks to perform complex tasks by learning patterns and representations directly from data. Unlike traditional machine learning approaches that require manual feature engineering, deep learning algorithms autonomously extract hierarchical features from data, leading to the creation of powerful and highly accurate models^18–20^. In this study, a CNN architecture is employed.

#### Convolution neural network

Convolutional neural networks represent a major breakthrough in deep learning and computer vision. These architectures are specifically designed to extract meaningful features from complex visual data, such as images and video. The inherent structure of the CNN, consisting of convolutional layers, pooling layers, and fully connected layers, mimics the ability of the human visual system to recognize patterns and hierarchical features. Convolutional layers use convolutional operations to detect local features, which are then progressively abstracted by pooling layers that condense the information. The resulting hierarchical representations are then fed into fully connected layers for classification or regression tasks. CNN have redefined the landscape of image recognition, achieving remarkable success in diverse domains ranging from image classification and object detection to face recognition and medical image analysis^21^.

### Transfer learning

Transfer learning stands as a fundamental concept within both machine learning and deep learning, involving the utilization of knowledge garnered from training a model on a particular task and subsequently applying that knowledge to another related task. In the realm of neural networks, transfer learning manifests significant potency. It encompasses the process of employing a pre-trained model, typically trained on a comprehensive and varied dataset, and fine-tuning it on a fresh dataset or task ^21–23^.

In this study, transfer learning models InceptionV3, VGG16, VGG19, and EfficientNetB4 were used in the classification process.

#### VGG

This architecture stands as a notable CNN model introduced by^24^, which builds upon its predecessor, the AlexNet model. It achieves this enhancement by replacing the initial 11 × 11 and 5 × 5 kernels in the first two convolutional layers with a series of consecutive 3 × 3 kernels. The model occupies approximately 528 MB of storage space and has achieved a documented top-5 accuracy of 90.1% on ImageNet data, encompassing approximately 138.4 million parameters. The ImageNet dataset comprises approximately 14 million images categorized across 1000 classes. The training of VGG16 was conducted on robust GPUs over the span of several weeks. This study used VGG16 and VGG19.

#### EfficientNET

EfficientNet is a family of scalable and efficient CNN models. The main goal of this series is to achieve better performance with fewer parameters. The term "EfficientNet" is a combination of the words "efficiency" and "network". The model series is mainly used in visual processing tasks such as image classification.

EfficientNet is a family of models that delivers competitive results in both performance and computational cost. It offers variations of different size and complexity at different scales. Higher numbered models are typically larger and more complex, but require more computing power. It was the top performing model in the ImageNet competition^24^.

#### Inception

The Inception architecture is an architecture used in the field of deep learning and CNN. It is designed to perform feature extraction and classification tasks more efficiently. First introduced in a paper titled "Going Deeper with Convolutions", the Inception architecture aims to provide better performance when processing complex visual datasets ^25^. The Inception architecture has a structure that includes parallel convolution layers and combines the outputs of these layers. In this way, features of different sizes can be captured and processed simultaneously^25^.

### Performance metric

Performance evaluation methods such as Accuracy, Precision, Recall, and F-score are used to evaluate models created for classification problems such as image processing. These methods are obtained from the confusion matrix. The confusion matrix is given in Table 2^26^.

**Table 2:** Confusion matrix.

	Actual value		
	Positive	Negative	Total
Estimate value			
Positive	${T}_{p}$	${F}_{p}$	TPos
Negative	${F}_{N}$	${T}_{N}$	TNeg
Total	Pos	Neg	M

In Table 2, the symbols ${T}_{N}$, ${T}_{P}$, ${F}_{P}$, and ${F}_{N}$ correspond to the true negative, true positive, false positive, and false negative values, respectively. From Eqs. (1) to (4), Accuracy, Precision, Recall and F-score is given respectively.

1 $$Accuracy=\frac{{T}_{P}+{T}_{N}}{{T}_{P}+{F}_{P}+{F}_{N}+{T}_{N}}$$

2 $$Precision =\frac{{T}_{P}}{TPos}$$

3 $$Recall=\frac{{T}_{P}}{Pos}$$

4 $$F{\text{-}}score=\frac{2*Precision*Recall}{Precision+Recell}$$

#### Receiver operating characteristic (ROC) curve

The ROC Curve is a graphical tool used to evaluate the performance of a classification model, particularly in binary classification scenarios. It provides a visualization of the sensitivity and specificity of the model, showing their variation as thresholds are changed ^27^. The ROC curve is plotted with the false positive rate on the x-axis and the True Positive Rate (TPR) on the y-axis. An optimal classifier, characterized by a TPR of one and a false positive rate of zero, lies in the upper left corner of the graph. The curve takes shape around this point, illustrating the performance of the model across different thresholds^26^.

In addition, the area under the receiver operating characteristic (ROC) curve, commonly referred to as the "area under the curve", succinctly summarizes the overall model performance in a single metric. The AUC value ranges from 0 to 1, with values closer to 1 indicating the increased discriminative ability of the model^26^. The ROC curve and AUC value serve as essential tools for comparing models and understanding classification model performance. A higher AUC value generally indicates superior model performance, while the curve illustrates the model's performance strengths and weaknesses at various thresholds^26^.

### Approval for participation

As the data is open source, there are no experiments on humans conducted by the authors. Open source has been studied on MRI images.

## Experimental settings and results

This study addresses the problem of image classification using deep learning methods. The most important and widely studied of these problems is that of health images. In this context, five different models (InceptionV3, EfficientNetB4, VGG16, VGG19, Multi-Layer CNN) were selected for the classification of brain tumors and their performances were compared on the same dataset. 10% of the dataset was used for testing, 15% for validation and 75% for training. All experimental setup and results were done at Google Colab.

### Multi-layer CNN

First, we need to determine the architecture of our model. The input form of our data is 400 × 400 and has 3 channels. Since we have a total of 4 different classes, the number of output classes is set to 4. Our model has a structure that includes convolutional and pooling layers. First, there is a 3 × 3 convolutional layer with 32 filters. This is followed by a 2 × 2 max pooling layer. This reduces the size by emphasizing lower-level features. To deepen our model, this structure is repeated twice, adding convolutional layers with 64 and 128 filters, respectively, and maximum pooling layers of size 2 × 2.

The resulting feature map is transformed into a flat vector with a flattening layer. A hidden (dense) layer of 128 neurons is then added. This layer deepens the learned features and increases generalization. Finally, the output layer has 4 neurons and calculates the probabilities between classes with the softmax activation function. To train our model, we need to determine the optimal function and metrics. In this paper, we use the Rectified Adam optimization algorithm. This algorithm dynamically adjusts the learning rate and helps to use gradients more efficiently. Also, categorical cross-entropy is used as the loss function during training, as it is widely used in multiclass classification task.

The metrics tracked during training are accuracy, as well as precision and recall. These metrics are important for evaluating the classification performance of the model. In addition, a reduced learning rate recall (ReduceLROnPlateau) is used to dynamically adjust the learning rate. This recall reduces the learning rate when the loss function flattens out during the training process, resulting in more stable training. The epoch is set to 14 and the batch size to 10.

### CNN-based transfer learning

In transfer learning architectures, all parameters and layers outside the model are the same, but after the last 3 layers of transfer learning models are removed, layers unique to the dataset are added instead: the GlobalAveragePooling2D layer contains fewer parameters than the Flatten layer, which reduces the risk of overfitting and helps build a more efficient model. Also, while the Flatten layer is used to organize the data, the GlobalAveragePooling2D layer is used for feature extraction, making the network learning process more efficient.

Due to the fact that the training data tends to learn very fast compared to the validation data, we modified the ratio of the dropout layers in the original architectures. For all models, the dilution rate was set to 0.05. During the model training process, the designated optimizer was "RectifiedAdam", with the optimizer parameters configured as follows: learning_rate = 0.0001, beta_1 = 0.9, beta_2 = 0.999, and epsilon = 1e−08. The loss function selected is categorical_crossentropy, while the metrics used include precision, recall, categorical accuracy, and accuracy. This completes the pre-training of the model. The final layer of the model is the dense layer, which contains 4 neurons, which is usually the number of output classes in classification problems. The activation function of this layer is "softmax". The softmax function makes the output values interpretable as probabilities between classes. Furthermore, the data type of this layer is "float64", which means that the output values are of a 64-bit double precision type. The layer also applies regularization using the "kernel_regularizer" property. The L2 regularization used here aims to reduce the risk of overfitting by limiting the size of the weights. The regularization coefficient of 0.1, denoted by "regulars. l2 (0.1)", controls the effectiveness of the regularization.

During the model training process, the "ReduceLROnPlateau" function of the Keras library was used as a backpropagation algorithm. This function automatically reduced the learning rate when the model approached a local optimum or when the loss value did not decrease. The parameters of the "ReduceLROnPlateau" function are as follows monitor: The metric monitored is usually "val_loss" (validation loss). This is the metric used to determine if the learning rate should be reduced:

- patience: The expected patience time for lowering the learning rate, i.e. how long the metric should not improve.
- factor: The factor used to reduce the learning rate. For example, a value of 0.3 reduces the learning rate by 30%.
- min_lr: Specifies the minimum achievable learning rate. This limits the learning rate without making it infinitesimal.

**Figure 2:**
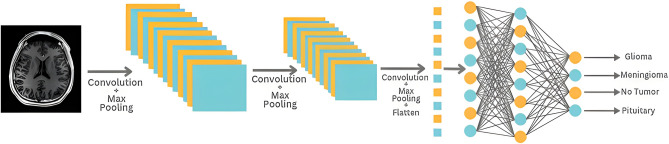
Multi-layer CNN model arthitecture.

Using this feature allows for more stable and efficient model training, streamlining the process of fine-tuning training parameters without the need to manually adjust the learning rate. The training program was run over 14 epochs with batches of size 10. Details of the multilayer CNN model used in the study are presented in Fig. 2, which outlines its architectural features.

**Figure 3:**
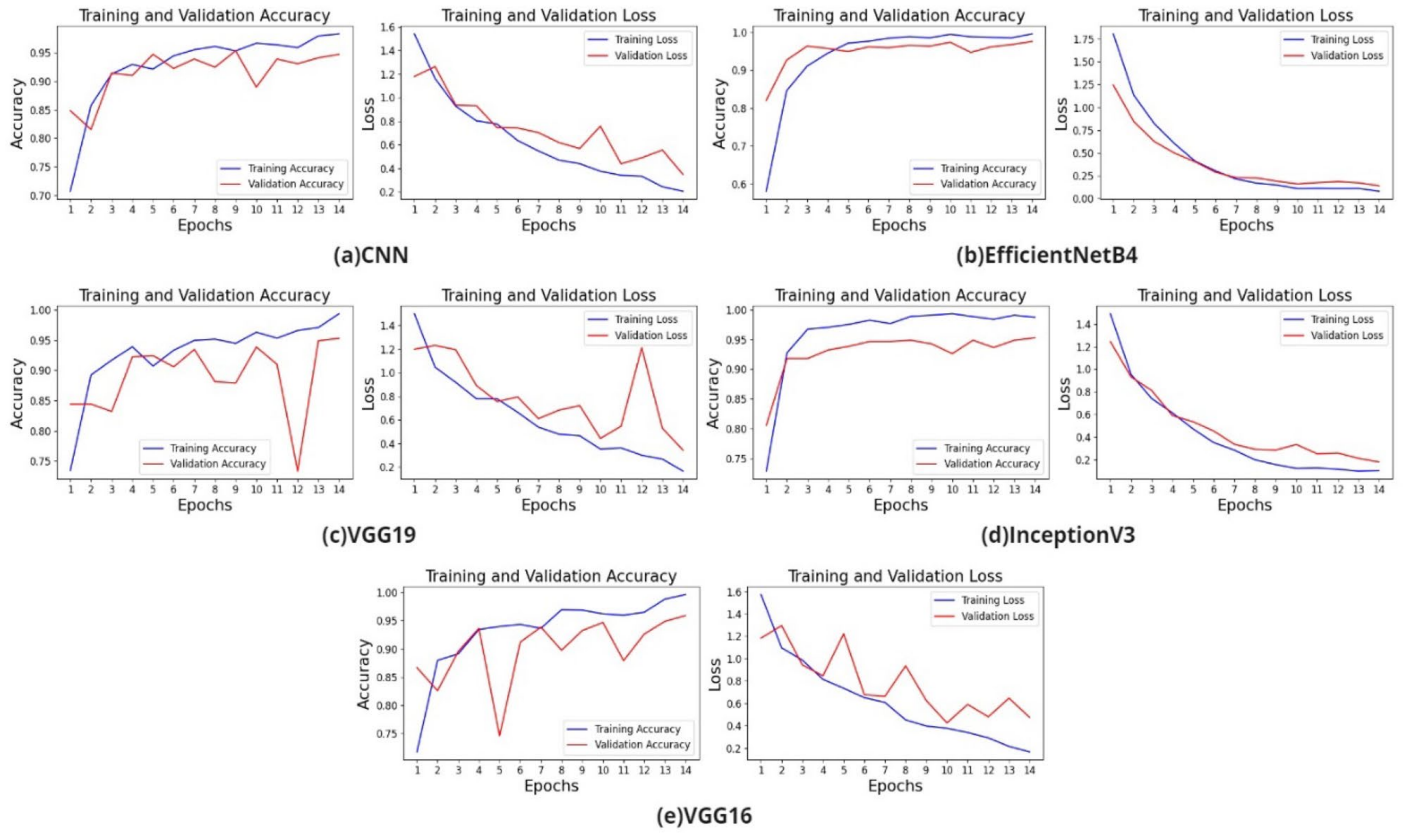
Learning curves of losses and accuracies of (**a**) CNN model, (**b**) EfficientNetB4 model, (**c**) VGG19 model, (**d**) InceptionV3, (**e**) VGG16 model.

The training and validation accuracy loss graphs of the models created with VGG19, EfficientNetB4, InceptionV3 transfer learning, and CNN are shown in Fig. 3.

Table 3 shows the accuracy, F-score, Recall, Precision and AUC results of the models created in the study.

**Table 3:** Performances (%) of the models created in the study.

Models	Accuracy	F-score	Recall	Precision	AUC
VGG19	96	96	96	96	99
EfficientNETB4	97	96	97	97	99
InceptionV3	96	96	96	96	99
3 CNN Model	91	90	91	91	98
VGG16	98	97	98	98	99

**Figure 4:**
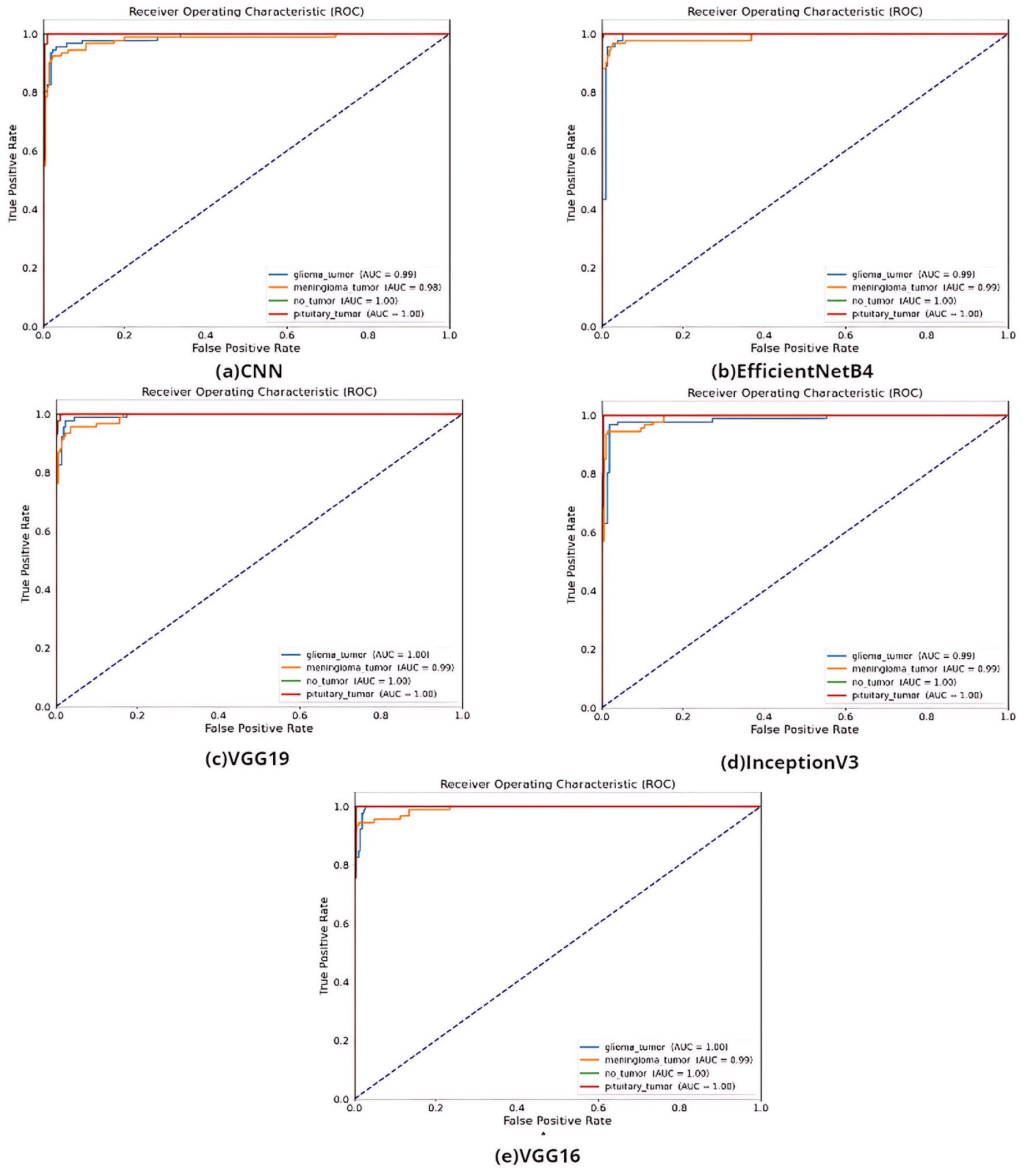
The ROC curve of (**a**) CNN model, (**b**) EfficientNetB4 model, (**c**) VGG19 model (**d**) InceptionV3 model, (**e**) VGG16.

According to Table 3, the best accuracy result was obtained by VGG16 with 97%. It is ahead of other methods with F-score value of 97%, AUC value of 99%, recall value of 98% and precision values of 98%. The ROC curves of the models created in the study are shown in Fig. 4.

**Figure 5:**
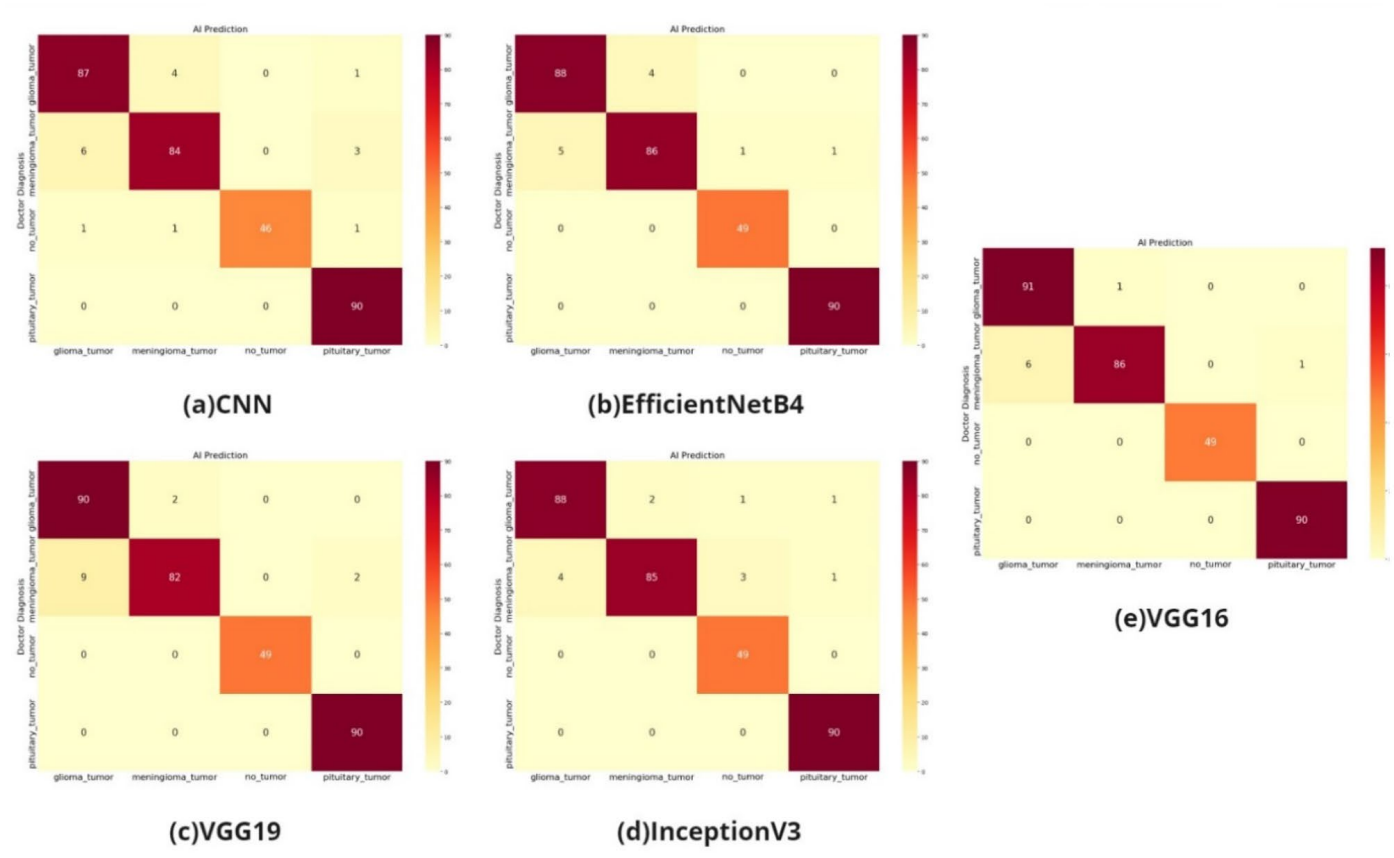
The confusion metrics of (**a**) CNN model, (**b**) EfficientNetB4 model, (**c**) VGG19 model (**d**) InceptionV3 model, (**e**) VGG16.

According to the AUC values in Fig. 4, the transfer learning models VGG, InceptionV3, and EfficientNetB4 and the models built with CNN have distinctive features. The confusion matrix of the study on the classification of glioma, meningioma, non-tumor normal patients, pituitary tumor patients in the dataset by tumor type is shown in Fig. 5.

As shown in the confusion matrix in Fig. 5, the classification performance is high for all four models (VGG16 and VGG19 models, CNN model, EfficientNetB4 model, InceptionV3 model).

## Results and discussion

As part of the study, CNN and CNN-based transfer learning models such as InceptionV3, EfficientNetB4, VGG19 were trained on open-source shared brain tumor patients. The best accuracy result was obtained with EfficientNetB4 with 95%. The comparison of the brain tumor studies with the literature is shown in Table 4.

**Table 4:** Comparison with previous studies on brain tumor.

Authors	Dataset	Models	Accuracy (%)
Wallis and Buvat ^1^	Brain Tumor Dataset^28^	SVM	74
Seere and Karibasappa^2^	Their own Brain Tumor dataset		85.32
Ortiz-Ramón et al.^3^	Their own Brain Tumor dataset		89.6
Gupta and Sasidhar^4^	MICCAI 2012 Challenge database^4^		87
Gumaei et al.^5^	Brain Tumor Dataset^28^	RELM	92.61
Shahajad et al.^6^	Kaggle brain dataset^17^	SVM	92
Vankdothu et al.^7^	Brain Tumor Dataset^17^	CNN, LSTM CNN-LSTM	CNN 89 LSTM 90.02 CNN-LSTM 92
Sirinivas et al.^8^	Brain Tumor Dataset^17^	InceptionV3 VGG16 ResNET50	InceptionV3 78 VGG16 96 ResNET50 95
Choudhury et al.^9^	Their own Brain Tumor dataset	CNN	96.08
Martini and Oermann^10^	Their own Brain Tumor dataset	CNN	93.09
Sarkar et al.^11^	Their own Brain Tumor dataset		91.03
Arunkumar et al.^12^	None	SVM, KNN	92.14
Zacharaki et al.^13^	Their own Brain Tumor dataset		88
Cheng et al.^14^	Their own Brain Tumor dataset		91.28
Paul et al.^15^	Their own Brain Tumor dataset	CNN	91.43
Afshar et al.^16^	Brain Tumor Dataset^28^	CapsNet	90.89
**This study**	Brain Tumor Dataset^17^	EfficientNetB4 InceptionV3 VGG19 VGG16 CNN	97 95 96 **98** 91

Best results obtained in the study.

As shown in Table 4, the CNN-based transfer learning models used in the study performed better. AI in healthcare plays an important role in the management of complex diseases such as brain tumors. AI enables faster, more accurate, and more effective diagnosis and treatment processes. However, AI technology is not intended to completely replace doctors, but to support and enhance their work. To realize the full potential of AI, it is important to consider issues such as ethics, security and privacy. In the future, AI-based solutions will continue to contribute to better management of brain tumors and other health problems, and improve the quality of life for patients. As seen in this study, AI-based studies will increase their importance to human health, from early diagnosis to positive progress in the treatment process.

Based on the results of this study, transfer learning methods should be preferred especially in image processing-based applications to support health decision makers. The data obtained from MRI or CT can be used as an early warning system to help health decision makers make quick and accurate decisions. Therefore, in addition to empirical analysis, AI-based applications should take a more active role as soon as possible. To this end, the diagnosis of diseases from instant CT or MR images will be investigated in the coming years.

## Limitation

With our motivation to investigate how it will work in single CNN and multilayer CNN based transfer learning models, we subjected the dataset to classification as it is without rotation and cropping operations, which is the most important limitation of our study.

## Data Availability

The dataset is shared open source. Availability Link: https://doi.org/10.34740/kaggle/dsv/1183165.
